# Clinical leadership during the COVID-19 pandemic: Reflections and lessons learned

**DOI:** 10.1177/08404704211044587

**Published:** 2021-10-25

**Authors:** Jonathan Sanders, Carl Balcom

**Affiliations:** 1 2757 Department of Emergency Medicine, HCA Houston Healthcare North Cypress, Cypress, TX, USA.; 2 16057 HCA Healthcare. Baylor University Louise Herrington School of Nursing, Dallas, TX, USA.

## Abstract

COVID-19 has, and continues to, wreak havoc worldwide, and the healthcare system has been particularly challenged with personnel shortages, resource insecurity, mixed messages, and fear to name a few. At the outset, it was thought the pandemic would be short-lived, resulting in the enactment of disaster plans in hospitals. Such autocratic approaches are not always effective in the long-term; a servant leadership approach is more conducive to engaging teams, and this dyad structure supports effective leadership during challenging times. While there is not one right approach to leading through a pandemic, lessons learned from this pandemic are applicable when, not if, the next pandemic occurs.

## Introduction

The COVID-19 pandemic has created devastation in every facet of life globally, and recovery will take years. The first cases of COVID-19 were reported in China in December 2019, and on March 11, 2020, the World Health Organization declared a global pandemic.^1,2^ It was originally thought that once the disease reached North America, hospital resources would be quickly overwhelmed; hospitals erected alternate care sites, cancelled elective activity, and enacted incident command systems for disasters, and so on.^3,4^

The healthcare system in particular has suffered in many different ways: mixed and conflicting messages, fluctuating patient volumes, resource uncertainty, and even personal illness and death. At the best of times, healthcare is a dynamic environment that requires its leaders to continually adapt their styles and approaches, and leading throughout the COVID-19 pandemic has put many to the test. The benefits of adopting a servant leadership approach in practice, along with a dyad leadership structure, will be explored through the use of autoethnography and examples of tactics that worked as well as lessons learned will be shared in this commentary. It is important to note that the authors’ approaches, experiences, and lessons learned must be considered contextually and applied accordingly, with due diligence in leadership practice.

## Background

It is universally accepted that good leadership is a necessary requirement in the healthcare sector. Effective leadership has been associated with improved quality of care and clinical outcomes (eg, pain management, restraint use, and indwelling catheter use); integrated care delivery; healthy work-practice settings for clinicians; and improved retention of staff.^5-8^ Many different leadership styles have been identified, all of which have advantages and disadvantages depending on the situation. A systematic review^9^ identified several leadership styles strongly correlated with improved outcomes in healthcare, including servant leadership. Servant leadership is a relationally driven leadership style based on the premise that the leader’s role is to serve their followers. This leadership style supports professional growth and development and team performance, and involves shared responsibility and authority, the use of active listening, empathy, and coaching for success.^9-11^

As healthcare organizations move toward inter-professional care and shared decision-making in patient care and program development, the traditional line-reporting structures are being replaced with dyad leadership structures, which have been associated with improved clinician engagement and team results.^12,13^ This leadership structure is particularly beneficial in the Emergency Department (ED) setting, where all health professionals, but in particular nurses and doctors, work side by side under some of the most challenging circumstances in the healthcare setting.

It should come as no surprise; therefore, that the presence of a dyad leadership structure (eg, medical and nursing/administrative) with both parties adopting a servant approach in their respective and collective practice would be well-suited to leading teams through a challenging event or period.

## Context

The authors both serve as emergency department leaders at busy community hospitals in the southern United States (US) and have previously worked together in a leadership dyad (medical and nursing/administrative). The authors consulted, and continue to consult, with one another regularly throughout the evolution of the COVID-19 pandemic to share tips, strategies, and lessons learned. This reflective commentary utilizes an autoethnographic approach to link lived experiences and approaches to leadership in managing the dynamic situation of the COVID-19 pandemic with established leadership practices. This approach leverages, examines, and makes inferences around the authors’ lived experiences, which supports and facilitates transferability and contextualization of the authors’ findings.^14,15^ While the authors’ experiences occurred within the U.S., the tactics, experiences, and lessons learned may be transferable to other settings.

## Leadership as a differentiator

As previously mentioned, a servant leadership style involves shared responsibility and the use of empathy as a leadership tool.^9^ This leadership style develops trust among followers and prevents feelings of isolation, antagonism (ie, “us against them”), and inequality, which in turn leads to enhanced performance.^16-18^ Examples of servant leadership practiced by the authors include the following:

- Regular one-on-one and group touch-points;
- Pre-emptive communication and information sharing to reduce anxiety around the unknown, or worse, around incorrect information;
- Timely resolution of followers’ concerns, such as ensuring the constant availability of Personal Protective Equipment (PPE); and
- Insulating clinician followers from the “noise” that was a constant, particularly in the initial months of the pandemic, so that they could practice and provide care without worrying about extraneous matters.

One of the practical benefits of an effective dyad leadership structure, where the two leaders are aligned in vision and behavior, is greater efficiency of both leaders: followers can approach either leader for support, and the mutual trust between the leaders allows the dyad to join forces and collaboratively rather than in parallel silos to improve the efficiency and expediency of leadership activities. For example, in the authors’ dyad structure both leaders were highly visible and accessible; either could be approached by followers indiscriminately; and both were trusted to resolve any issues that arose. In less effective dyad structures that the authors have been part of, situations were made more challenging due to the lack of alignment, inconsistency in messaging, and the ability of followers to undermine individual leader efforts. This results in increased workload for both leaders not only because of the loss of efficiency but also by having to spend time on rework and undoing damage.

## Effective leadership in the virtual realm

At the outset of the COVID-19 pandemic, physical distancing was one of the tactics implemented to help curb the spread of the virus. This included, among other things, cancelling in-person meetings and transitioning to virtual platforms as the primary source of team communication and interaction. The availability of technology was definitely a support to effective leadership, and it would be unimaginable not to have virtual communication platforms (eg, group messaging apps and virtual meeting platforms) at our disposal. Joslin and Joslin^19^ shared some examples of tactics used to maintain communication and support leadership, including frequent team huddles, daily information sharing on conference calls and virtual meetings, and virtual town halls. Both authors utilized these tactics with good results and increased the frequency of communication according to the level of anxiety among followers, both internal and external “noise,” and the availability of new evidence-based information.

The pandemic also forced the healthcare system and its leaders to reexamine the role of technology and telemedicine. Until the pandemic, telemedicine as a clinical tool was not as widely utilized as possible and was not seen as a comparable option for many payors. The pandemic forced its utilization, and in turn changes in government and other payor regulations, and now tele-health is a useable technological option.^20,21^ From the non-clinical end, the pandemic demonstrated that remote work and virtual meetings were just as effective, and in some cases employee productivity improved through remote work.^22^ To that end, these authors saw a significant reduction in meetings and committees that prior to the pandemic were deemed essential, with no concomitant deterioration in efficiency; feedback from followers regarding the increased visibility and availability of leaders was all highly favorable and in fact the reduction in meetings facilitated the use of servant leadership.

## Lessons learned

The COVID-19 pandemic took the world by surprise and has left drastic changes on not only the healthcare system but also on daily life as we knew it. Despite the fact that the pandemic is ongoing and its effects continually evolving, there are several “lessons learned” from the authors’ perspective.

We responded to the pandemic as a short-term disaster rather than a fundamental change to our world and way of living; no one had any idea of the devastation and turmoil that would follow and that is continuing even today. Further compounding this problem was the fact that information and directives were constantly changing, particularly from government agencies and other authorities, which resulted in mistrust across the board.^19^ Also, because the initial approach to managing the pandemic was based on the disaster model and so incident command-type structures were set up. This approach follows a command-and-control structure using an autocratic process for decision-making and communication. The next time, it would be more beneficial to incorporate a process and workflow reengineering perspective and, in keeping with the principles of servant leadership and shared governance, engage frontline clinicians and leaders in planning around service delivery, PPE utilization, staffing, communication strategies, and so forth.^23^ While the authors do not negate the valuable role that incident command and autocracy play in managing a crisis situation, the importance of ensuring that healthcare professionals feel listened to and are made to feel part of the solution cannot be overemphasized. The authors also point out that consultation and timeliness are not mutually exclusive.

We also realized that there needed to be an increased focus on the emotional health and well-being of clinicians. For example, Kuhl et al.^24^ surveyed a sample of physicians during the initial stages of the pandemic and found high numbers of respondents experiencing symptoms of burnout; almost one quarter of respondents reported feeling that the state of their practice environments was forcing them to make ethical compromises. While emotional well-being has always been a concern of healthcare leaders, its importance came to the forefront during the pandemic. Some strategies that were reported to be effective in improving emotional health were spiritual programs (eg, chaplain use, prayer, yoga, and Zen practice at work) as well as enhanced leadership basics of communication, frequent employee rounding, and facilitation of peer support.^19^ Ensuring that clinicians had adequate time to decompress was a key priority for the authors; strategies included ensuring adequate rest between shifts, limiting the number of consecutive shifts, proactively touching base at the end of a challenging shift, and encouraging the taking of time off despite the possibility of traveling for leisure. Leadership presence and visibility were easier at the outset of the pandemic because certain routine activities (eg, meetings and regular reports) came to a halt; this allowed leaders to spend more time with their teams and increase their visibility. The significant restrictions on movement, lockdowns, and physical distancing measures affecting all persons further compounded the stress being placed on healthcare professionals.^25^

At the outset of the pandemic, and sporadically since, outpatient and elective activities have been cancelled or curtailed.^26^ In addition to the logical assumption that cancelling elective activities will eventually result in the need becoming emergent, there were significant financial consequences on health service providers. The majority of government funding was allocated to hospitals; clinicians involved in providing outpatient and elective services (eg, surgeons, primary care providers, and surgical nurses) as well as hospital-based clinicians with volume-driven income (eg, the ED) suffered significant loss of income which added additional stress and negatively impacted clinicians’ emotional well-being.^19,26,27^

Joslin & Joslin^19^ reported the four key challenges identified by nurse executives across the United States: implementing and communicating policy changes; being able to flex up staffing to address volume and acuity surges; ensuring emotional health of clinicians as well as access and availability of PPE and other medical equipment. These challenges are transferable system-wide and should be used as the basis in developing a pandemic playbook at all levels.

## Conclusion

The COVID-19 pandemic took the world by a storm and clearly showed that we as a healthcare system were not prepared: the lack of a unified plan, the disjoined and individualized responses by various jurisdictions, the lack of inventory control (eg, PPE and ventilators), and the lack of standardized disease surveillance data are just some of the indications that we were not prepared.^28^

Like any other disaster or crisis situation, leadership (or lack thereof) is truly the differentiator in how groups and entities fared. Utilizing the principles of servant leadership supports the emotional well-being and fosters engagement and trust among clinicians. Applying this leadership style in a dyad leadership structure promotes team unity while also supporting more efficient planning and execution of plans and tactics. A collaborative and unified approach between the two leaders is a necessary requirement for success. As mentioned, the authors do not dispute the need for decisive decision-making during a disaster but contend that there is an opportunity to be consultative and follow the principles of servant leadership without creating undue delay. The science of leadership involves understanding accepted theories and best practices; the art of leadership involves the skillful and intentional application taking into consideration the context of a leader’s practice.

There have been many lessons learned throughout the pandemic, and as we move into our “new normal,” continuing to support and place emphasis on the emotional health and well-being of clinicians is a requirement. In addition to the many professional challenges, the COVID-19 pandemic has caused personal harm to many healthcare professionals and leaders; the authors have both experienced personal and familial suffering and even loss throughout the waves of the pandemic, including at the time of this article being finalized. Engaging and supporting clinicians through empathy, shared decision-making, and inter-professional collaboration will be the differentiator.
